# The effect of screw orientation on internal fixation of Letenneur type II Hoffa fractures: a biomechanics study

**DOI:** 10.1186/s12891-024-07222-6

**Published:** 2024-02-02

**Authors:** Jialun Liu, Zhe Lu, Zhanle Zheng

**Affiliations:** Department of Orthopaedics, Third Hospital of Hebei Medical University, Shijiazhuang, Hebei Province China

**Keywords:** Hoffa fracture, Nail orientation, Biomechanics, Internal fixation

## Abstract

**Background:**

To investigate the biomechanical effects of screw orientation and fracture block size on the internal fixation system for Letenneur type II Hoffa fractures.

**Methods:**

The fracture models were randomly divided into six groups according to the fracture subtypes and the direction of nail placement, and a plumb line of the posterior condylar tangent was made across the base of the posterior femoral condyle. The fracture blocks of the three types of fracture were calculated and recorded in the sagittal position, and the biomechanical performance of the six groups was evaluated by biomechanical tests. The axial load on the fracture block at a displacement of 2 mm was set as the failure load, a gradually increasing axial load was applied to each fracture model using a customized indenter at a load of 250-750 N, and the displacements and failure loads of the six groups were recorded at different axial loads.

**Results:**

Biomechanical test results showed that the larger the fracture block, the greater was the stability when nailing from front to back, and the smaller the fracture block, the greater was the strength when nailing from back to front (*p* < 0.001). As the fracture block became larger, the biomechanical advantage of nailing from posterior to anterior decreased.The displacement under 250 N load were 1.351 ± 0.113 mm, 1.465 ± 0.073 mm for Group IIa AP and Group IIa PA. The displacement under 500 N load were 2.596 ± 0.125 mm, 2.344 ± 0.099 mm for Group IIa AP and Group IIa PA. The displacement under 750 N load were 3.997 ± 0.164, 3.386 ± 0.125 mm for Group IIa AP and Group IIa PA. The failure loads were 384 ± 14 N, 415 ± 19 N for Group IIa AP and Group IIa PA. In the type IIa fracture group, the difference was no longer significant (*p* > 0.001). Therefore, there is a mechanical threshold that ranges from 38.36 to 52.33% between type IIa and type IIb fractures.

**Conclusions:**

The effect of the nailing direction on the strength of fixation has a fracture-block critical point, which is consistent overall with the trend that the larger the fracture block is, the greater the stability when nailing from anterior to posterior, and the smaller the fracture block is, the greater the strength when nailing from posterior to anterior.

**Supplementary Information:**

The online version contains supplementary material available at 10.1186/s12891-024-07222-6.

## Background

Hoffa fractures are fractures on the coronal plane of the distal femur and account for approximately 0.1% of all fractures in the body [1, 2]. Hoffa fractures, as intra-articular fractures, require anatomical reduction and strong fixation [3, 4]. Screw fixation is mostly used for simpler Hoffa fractures [5, 6]. There are 2 directions of nail placement, which are from front to back and from back to front [7]. Clinically, the direction of nailing is generally chosen according to the size of the fracture block. When the fracture block is large, anterior-to-posterior nailing is used, which can reduce the intrusion to the rich soft tissues in the posterior part of the knee joint and reduce the incidence of medical injury; however, when the fracture block is small, anterior-to-posterior nailing is more difficult to perform, and posterior-to-frontal nailing is predominantly used.

The direction of Hoffa fracture pinning is mostly based on the surgeon’s clinical experience; however, a biomechanical approach may shed new light on this topic. Internal fixation of Hoffa fractures requires strong biomechanical strength [8, 9]. Scholars have found that screws placed from posterior to anterior are significantly stronger and more stable for fracture fixation than those placed from anterior to posterior [10, 11]. However, in these experiments, the biomechanical experiments were performed with uniform modeling [12], i.e., the fracture position was fixed, and then biomechanical experiments were performed with different nail placement directions. One variable that was overlooked was the size of the fracture block. If the size of the fracture block is changed and biomechanical experiments in different directions are performed, the results may be different. Therefore, we performed fixation with different nail placement directions on models with fracture blocks of different sizes and performed an all-around comparison.

## Methods

### Ethics statement

This study was approved by the Ethics Committee of the Third Hospital of Hebei Medical University(K2015-001-12). All methods were carried out in accordance with the guidelines (Helsinki Declaration) for biomedical research. All materials, data and associated protocols in this study are available to readers and the publishing team.

### Fracture model construction and fixation

In this study, a total of 48 intact adult embalmed knee specimens were used to create Hoffa fracture models. Fracture, tumor, deformity, and severe osteoporosis were ruled out from all specimens by the naked eye and X-ray photography.

#### Models of Hoffa fractures

**Fig. 1:**
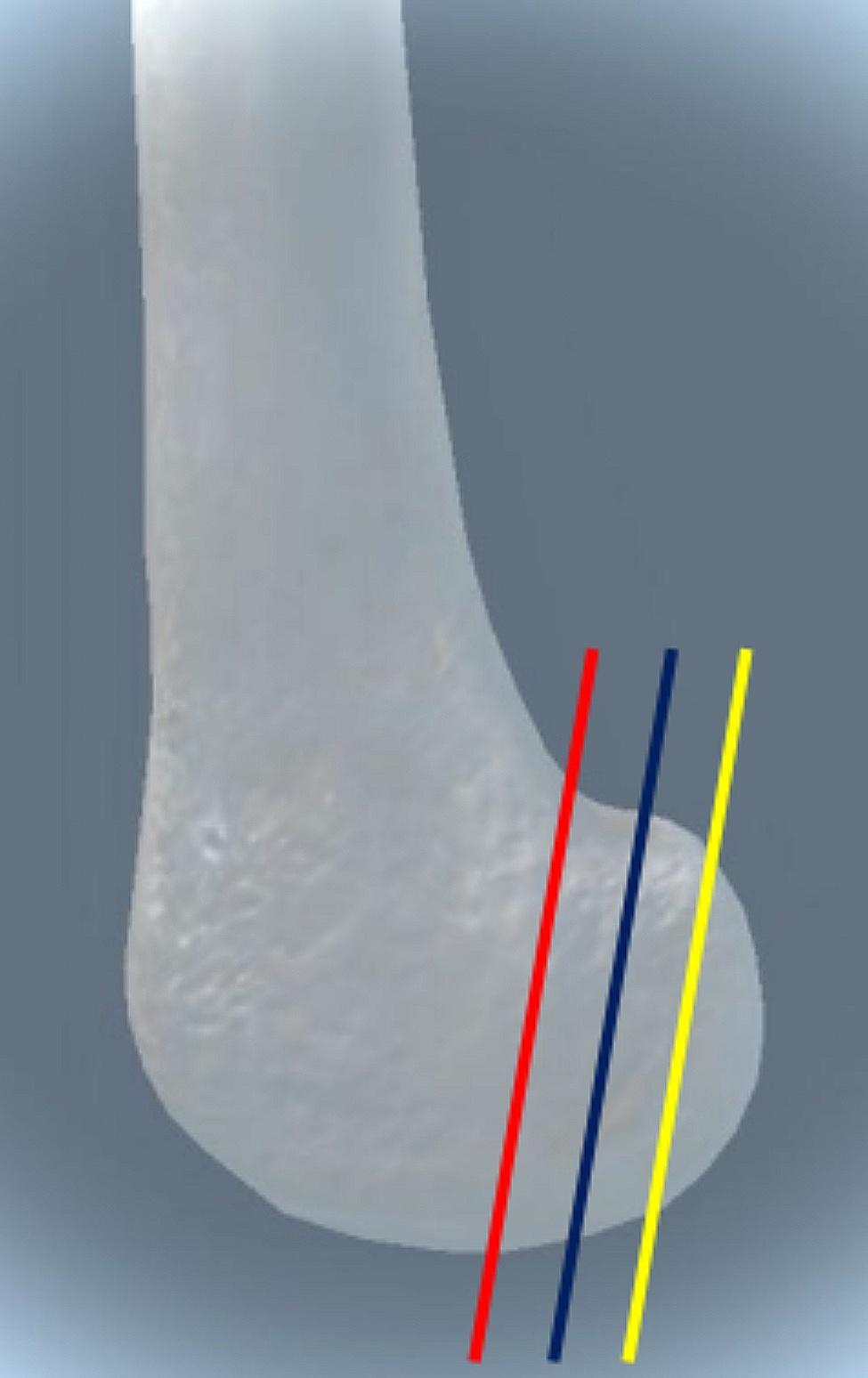
Different osteotomy positions for Letenneur type II fractures

All specimens preserved only the lower 20 cm of the femur, and the skin, subcutaneous and other soft tissues were eliminated as much as possible (marking the osteotomy position before elimination). Only the femur was preserved, and the Letenneur fracture model of Letenneur type II was fabricated by using the Letenneur typing system as a reference [13](Fig. 1). In addition, all preparations were performed by a single surgeon.

#### Fracture fixation groups

**Fig. 2:**
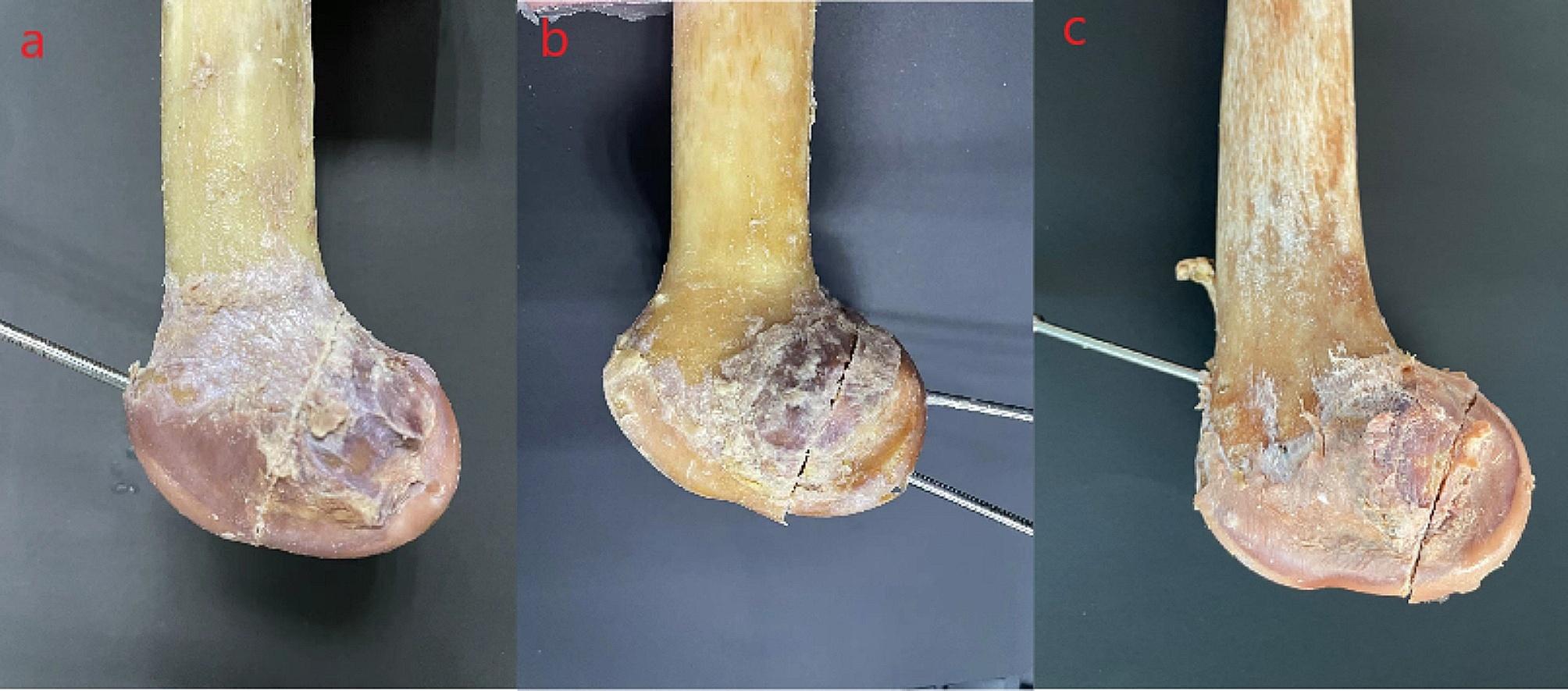
Letenneur type II Hoffa fracture subtypes.**(a)**Group IIa. **(b)**Group IIb. **(c)**Group IIc.

**Fig. 3:**
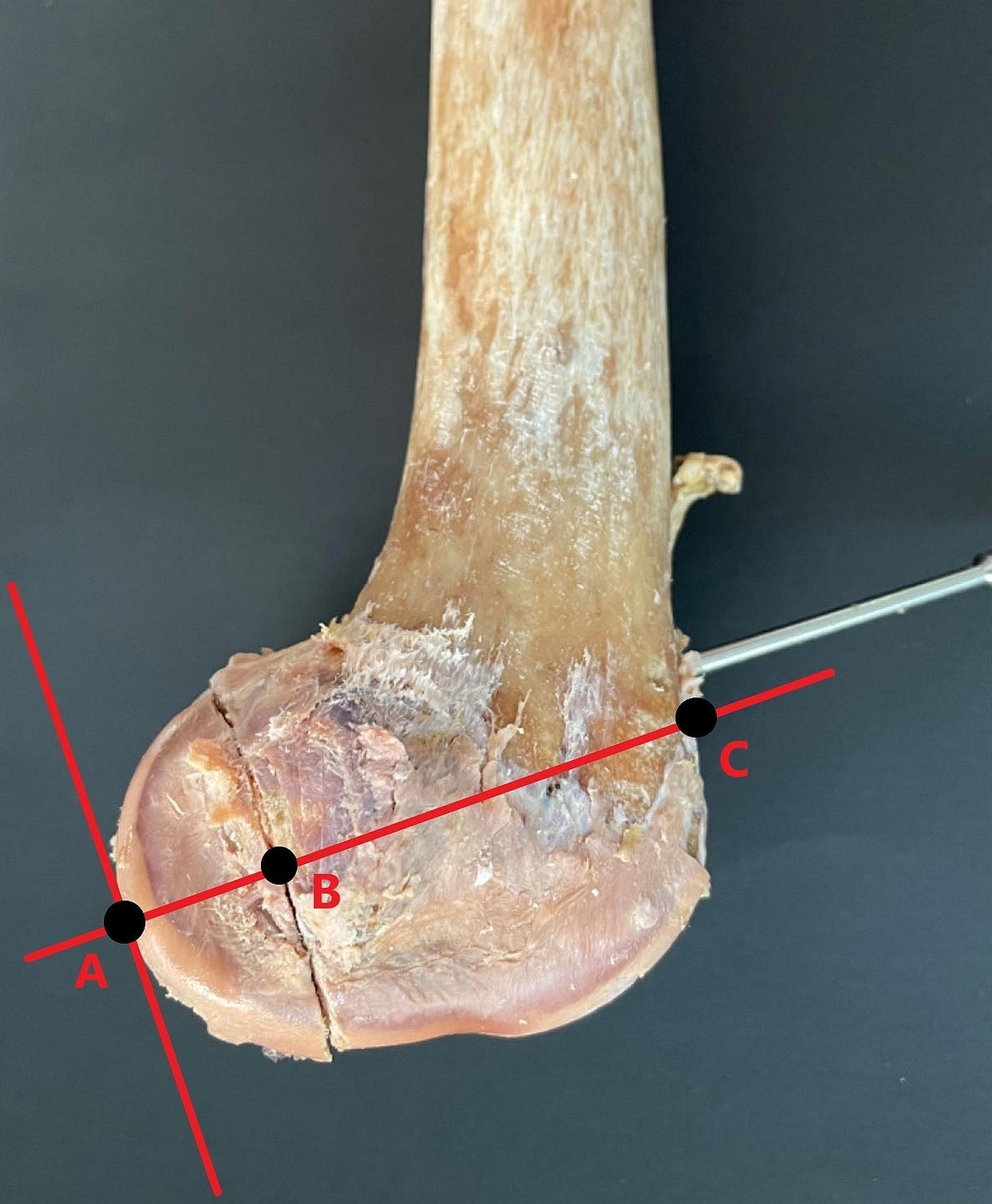
Percentage of bone mass in the sagittal position (AB/AC)

The fracture subtypes were categorized and grouped into the type IIa group, type IIb group and type IIc group (type IIa fracture block: with complete gastrocnemius or popliteal tendon attachment, type IIb fracture block: with partial gastrocnemius or popliteal tendon attachment, and type IIc fracture block: without ligamentous attachment) (Fig. 2), with 16 cases in each group. A plumb line of the posterior condylar tangent was made across the base of the posterior femoral condyle, and the percentage of the bone mass in the sagittal position for the three types of fractures was calculated, recorded and averaged (Fig. 3). All fractures were fixed in parallel with two screws, and each group was divided into two groups of eight cases each according to the direction of nail placement, so that we had a total of six groups: IIa AP, IIa PA, IIb AP, IIb PA, IIc AP, and IIc PA.

### Biomechanical testing

**Fig. 4:**
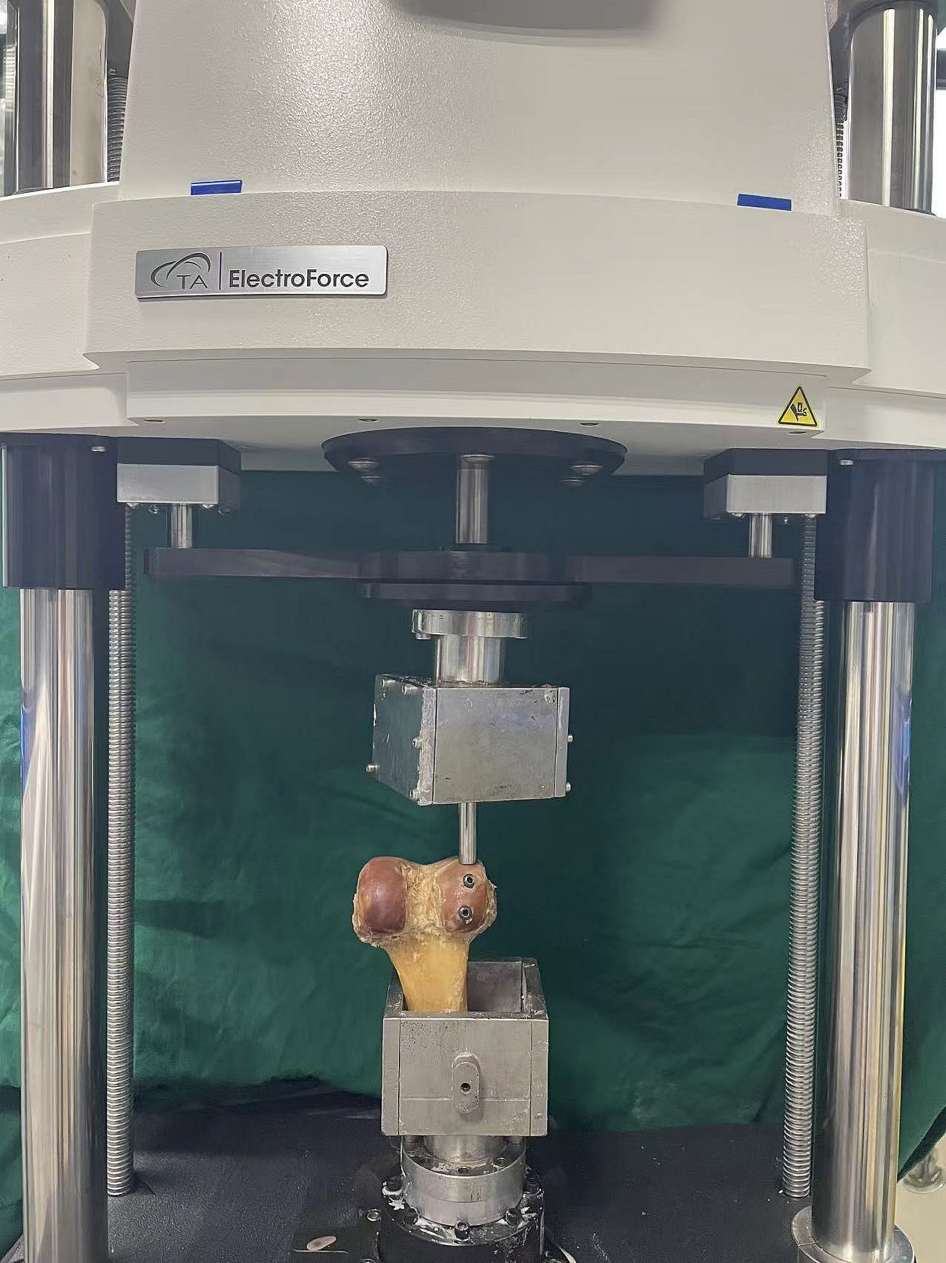
The biomechanical machine(Medium-sized biomechanical machine: ELF3300 test system)

Each fracture model was placed vertically inverted into the biomechanical tester and secured with dental powder, and the load was applied to the fracture block by means of a customized cylindrical pressurizer (Fig. 4). During normal gait, the biomechanical load on the knee joint is approximately 2–3 times the body weight, with the medial and lateral loads being approximately 55% and 45%, respectively, and the forces are reciprocal; therefore, when the human body weight was set at 60 kg, we chose three different axial peak loads, 250 N, 500 N, and 750 N (1–3 times the body weight), to simulate the forces in the case of a single-leg stance. After each fracture model was mounted, a progressively increasing axial load was applied to each model at a loading rate of 10 N/s. The axial load was applied to each model at a loading speed of 10 N/s. The axial displacement from the initial position to the peak axial load was continuously captured using Bluehill software. In addition, the failure load was set to the load at displacements up to 2 mm. Finally, the displacements and failure loads at the three loads were recorded to evaluate the biomechanical stability of the three different internal fixations.

### Statistical analysis

All statistical data in our study were analyzed using SPSS software (version 26.0; SPSS Inc., Chicago, IL, USA). Continuous variables are presented herein as the mean values and standard deviation (SD) and were determined using independent-sample Student t tests or Mann–Whitney U tests depending on whether the values of the variable were normally distributed. In addition, continuous data with skewed distributions were categorized by reference values. The significance level was set at *P* < 0.001.

## Results

**Fig. 5:**
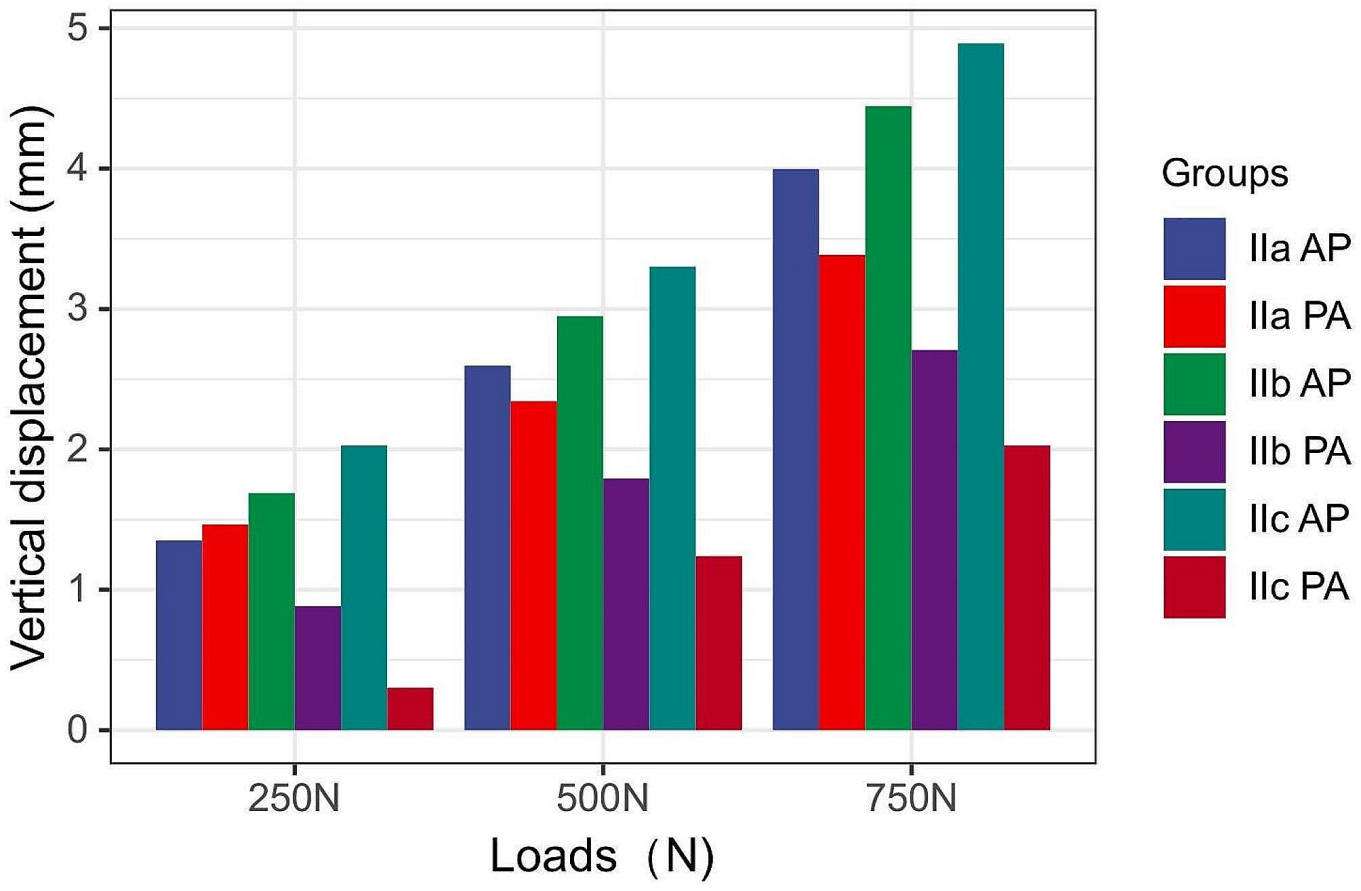
The vertical displacement of the fragment under three different axial loads. The values are mean displacements (measured in mm)

**Fig. 6:**
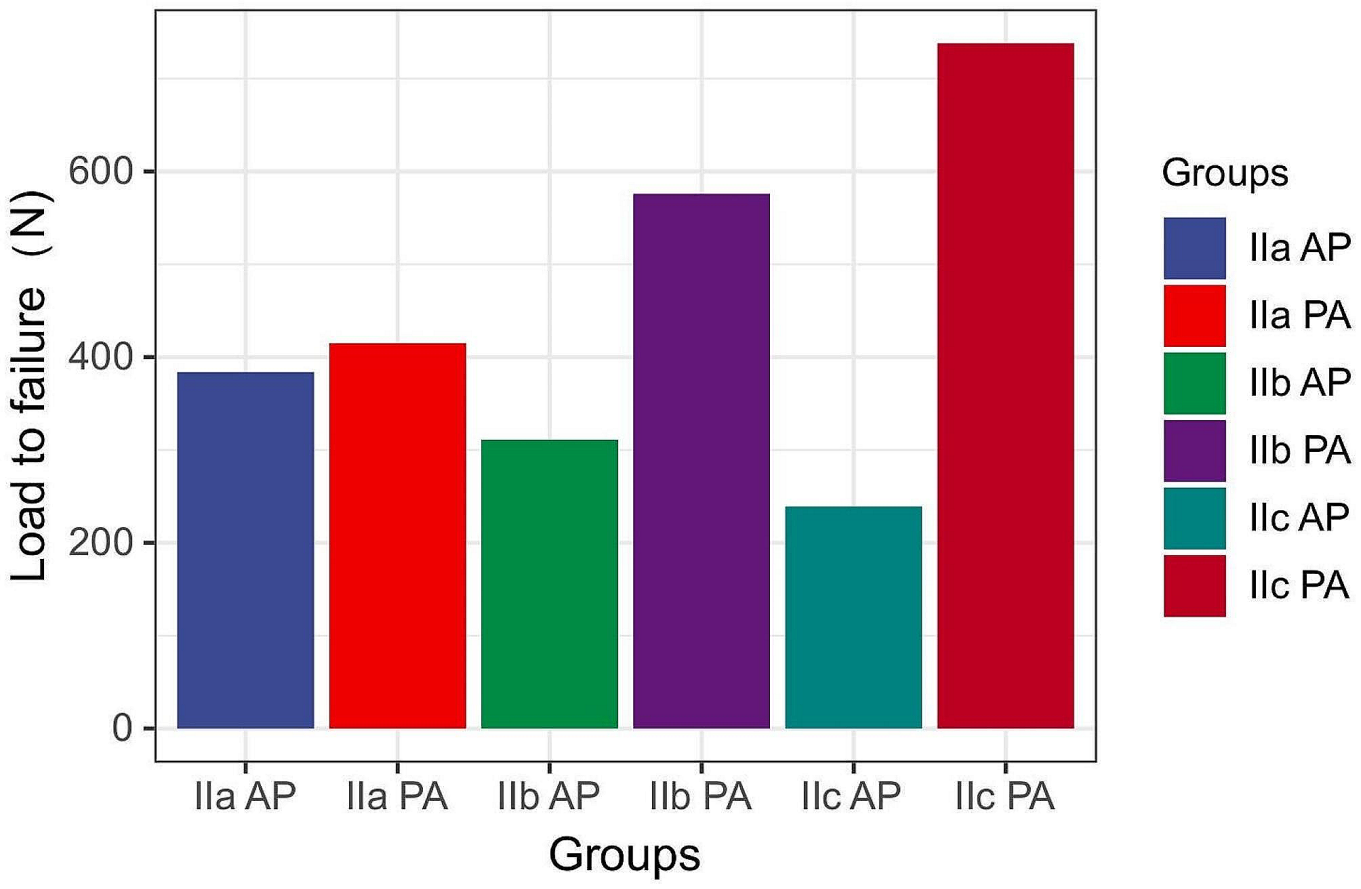
Failure loads of the six groups. The values are mean loads (measured in N)

Table 1 shows the vertical displacement (Fig. 5) and failure load (Fig. 6) of the fracture blocks in each group under different axial loads, and Table 2 shows the percentage of the bone blocks in each group in the sagittal position. The larger the fracture block is, the greater the stability when nailing from anterior to posterior, and the smaller the fracture block is, the greater the strength when nailing from posterior to anterior (*p* < 0.001). As the fracture block became larger, the biomechanical advantage of nailing from posterior to anterior decreased.The displacement under 250 N load were 1.351 ± 0.113 mm, 1.465 ± 0.073 mm for Group IIa AP and Group IIa PA.The displacement under 500 N load were 2.596 ± 0.125 mm, 2.344 ± 0.099 mm for Group IIa AP and Group IIa PA.The displacement under 750 N load were 3.997 ± 0.164, 3.386 ± 0.125 mm for Group IIa AP and Group IIa PA.The failure loads were 384 ± 14 N, 415 ± 19 N for Group IIa AP and Group IIa PA.In the type IIa fracture group, the difference was no longer significant (*p* > 0.001). Therefore, there is a mechanical threshold that ranges from 38.36 to 52.33% between type IIa and type IIb fractures.

**Table 1:** Vertical displacement of the Hoffa fracture at three different load levels and from load to failure

Groups	Vertical displacement (mm)			Load to failure (N)
	250 N	500 N	750 N	
IIa AP	1.351 ± 0.113	2.596 ± 0.125	3.997 ± 0.164	384 ± 14
IIb AP	1.689 ± 0.116	2.949 ± 0.116	4.445 ± 0.196	311 ± 13
IIc AP	2.028 ± 0.119	3.302 ± 0.106	4.892 ± 0.230	239 ± 14
IIa PA	1.465 ± 0.073	2.344 ± 0.099	3.386 ± 0.125	415 ± 19
IIb PA	0.884 ± 0.069	1.792 ± 0.095	2.708 ± 0.108	576 ± 17
IIc PA	0.303 ± 0.065	1.240 ± 0.091	2.029 ± 0.091	738 ± 19
P (IIa AP-IIb AP)	0.000	0.000	0.000	0.000
P (IIa AP-IIc AP)	0.000	0.000	0.000	0.000
P (IIb AP-IIc AP)	0.000	0.000	0.001	0.000
P (IIa PA-IIb PA)	0.000	0.000	0.000	0.000
P (IIa PA-IIc PA)	0.000	0.000	0.000	0.000
P (IIb PA-IIc PA)	0.000	0.000	0.000	0.000
P (IIa AP-IIa PA)	0.031	0.001	0.000	0.002
P (IIb AP-IIb PA)	0.000	0.000	0.000	0.000
P (IIc AP-IIc PA)	0.000	0.000	0.000	0.000

IIa Letenneur type IIa fracture, IIb Letenneur type IIb fracture, IIc Letenneur type IIc fracture, AP anterior to posterior nailing, PA posterior to anterior nailing

**Table 2:** Percentage of bone mass in the sagittal plane for each group

Groups	Percentage of bone mass in sagittal position (%)
IIa	52.33
IIb	38.36
IIc	26.84

IIa Letenneur type IIa fracture, IIb Letenneur type IIb fracture, IIc Letenneur type IIc fracture

## Discussion

Hoffa fracture is an intra-articular fracture that requires high-quality surgery [14]. The main points of surgical treatment are as follows: (1) Anatomical repositioning is necessary [15]. (2) Strong fixation is required [16, 17]. (3) Since the fracture block has fewer soft tissue connections and poorer blood flow, to avoid ischemic necrosis, stripping of the periosteum should be minimized [18]. (4) Hoffa fracture is often accompanied by tendon and ligament attachment point injury, and clinicians should focus on soft tissue repair to further promote the recovery of knee joint stability and function [19–22]. (5) Screw tails are recommended to be buried for treatment [3].

Some Hoffa fractures can be fixed with screws, clinically, there are two main directions to choose from: front-to-back nailing and back-to-front nailing [23, 24]. Generally, the choice is based on the size of the fracture block, and when the fracture block is large, front-to-back nailing is usually chosen, which is related to the anatomical structure of the distal femur. The soft tissue coverage of the distal femur in the anterior part of the distal femur is less than that in the posterior part of the distal femur, and front-to-back nailing is helpful to reduce the surgical invasion of the soft tissue. This is confirmed by surgical access for incisional reduction. When the fracture mass is small, the surgical difficulty of anterior-to-posterior nailing is significantly higher, as it is difficult to drive the nail head into the fracture mass, and the fracture mass is not stable during the nailing process. Therefore, posterior-to-anterior nailing is usually used to expose the fracture block while providing stable temporary fixation of the fracture block, and screws are used for compression fixation.

In our study, the data showed that the larger the fracture block was, the greater the stability when nailing from anterior to posterior, and the smaller the fracture block was, the greater the strength when nailing from posterior to anterior (*p* < 0.001). As the fracture block became larger, the biomechanical advantage of nailing from posterior to anterior decreased, and in the type IIa fracture group, the difference was no longer significant (*p* > 0.001). Therefore, there is a mechanical threshold that ranges from 38.36 to 52.33% between type IIa and type IIb fractures.When the size of the fracture block is at or above the threshold, the difficulty of the operation and trauma to the soft tissues and joint surfaces should be taken into consideration for nailing, and biomechanical effects need not be taken into account. When the fracture block size is below the threshold, biomechanical effects should be taken into consideration.

These experimental data also have guiding significance for fractures such as posterior ankle fractures. When applying screws for fixation of single-block fractures, if the direction of nail placement does not have a significant effect on biomechanical strength, the following conditions need to be considered: (1) safety of access, (2) difficulty of manipulation, (3) invasion of soft tissues, (4) protection of cartilage, and (5) trade-offs for combining other fractures.

There are several limitations to this study. First, the sample size of this study was relatively small and could be further refined between the Type IIa and Type IIb groups. Second, the biomechanical evaluation of this study was relatively simple, and this experiment did not address the factors of knee stress and stability, including ligaments, muscles, and other soft tissues. It is hoped that these aspects will be improved in future studies.

## Conclusions

Our study revealed that there is a fracture block threshold for the effect of the nailing direction on fixation strength, which conforms overall to the trend of greater stability with larger fracture blocks when nailing from anterior to posterior and greater strength with smaller fracture blocks when nailing from posterior to anterior.

### Electronic supplementary material

Below is the link to the electronic supplementary material.

Supplementary Material 1

## Data Availability

All data generated or analyzed during this study are included in this published article [and its supplementary information files].

## References

[1] Zhang YZ (2014). Clinical epidemiology of orthopedic trauma.Fracture of tibia and fibula.

[2] Gavaskar AS, Tummala NC, Krishnamurthy M (2011). Operative Management of Hoffa Fractures–A prospective review of 18 Patients[J]. Injury.

[3] Patel PB, Tejwani NC (2018). The Hoffa fracture: coronal fracture of the femoral condyle a review of Literature[J]. J Orthop.

[4] Lu B, Zhao S, Luo Z (2019). Compression screws and buttress plate Versus Compression screws only for Hoffa fracture in Chinese patients: a comparative Study[J]. J Int Med Res.

[5] Hak DJ, Nguyen J, Curtiss S (2005). Coronal fractures of the distal femoral condyle: a biomechanical evaluation of four internal fixation Constructs[J]. Injury.

[6] Liebergall M, Wilber JH, Mosheiff R (2000). Gerdy’s Tubercle Osteotomy for the treatment of coronal fractures of the lateral femoral Condyle[J]. J Orthop Trauma.

[7] Sun H, He QF, Huang YG (2017). Plate fixation for Letenneur Type I hoffa fracture: a biomechanical Study[J]. Injury.

[8] Gammon L, Hansen E, Cheatham S (2020). Technique for reduction and fixation of a Hoffa Fracture with Ipsilateral Patella dislocation from low-energy trauma, a Rare Injury: a Case Report[J]. Jbjs Case Connect.

[9] Nandy K, Raman R, Vijay RK (2015). Non-union coronal fracture femoral condyle, sandwich technique: a Case Report[J]. J Clin Orthop Trauma.

[10] Yao SH, Su WR, Hsu KL (2020). A Biomechanical comparison of two screw fixation methods in a Letenneur type I hoffa Fracture[J]. Bmc Musculoskelet Disord.

[11] Soni A, Sen RK, Saini UC (2012). Buttress plating for a Rare Case of Comminuted Medial Condylar Hoffa Fracture Associated with Patellar Fracture[J]. Chin J Traumatol.

[12] Jarit GJ, Kummer FJ, Gibber MJ (2006). A mechanical evaluation of two fixation methods using Cancellous screws for coronal fractures of the lateral condyle of the distal femur (ota type 33B)[J]. J Orthop Trauma.

[13] Letenneur J, Labour PE, Rogez JM (1978). [Hoffa’s fractures. Report of 20 cases (author’s transl)][J]. Ann Chir.

[14] Zhang P, Zhang XZ, Tao FL (2020). Surgical Treatment and Rehabilitation for Hoffa Fracture Nonunion: two case reports and a literature Review[J]. Orthop Surg.

[15] Chang JJ, Fan JC, Lam HY (2010). Treatment of an osteoporotic Hoffa Fracture[J]. Knee Surg Sports Traumatol Arthrosc.

[16] Agarwal S, Krishna LG, Agarwalla A (2021). Lateral buttress plate along with Cancellous Screw fixation for Hoffa fracture using Swashbuckler Approach[J]. Indian J Orthop.

[17] Liu ZH, Wang T, Fang C (2021). Reverse contralateral proximal tibial plating and cannulated screws fixation for Hoffa fracture: a Case Report[J]. Trauma Case Rep.

[18] Chouhan D, Hooda A, Rana A (2020). Non-union lateral femoral Condyle Hoffa fracture: a Case Report[J]. Int J Burns Trauma.

[19] Lewis SL, Pozo JL, Muirhead-Allwood WF (1989). Coronal fractures of the lateral femoral Condyle[J]. J Bone Joint Surg Br.

[20] Maheshwari V, Sharma SL, Goyal D (2019). Clinical experience with management of Hoffa Fractures Using Headless Compression Screw and Headed Screw[J]. J Clin Orthop Trauma.

[21] Zhou Y, Pan Y, Wang Q (2019). Hoffa Fracture of the femoral condyle: Injury mechanism, classification, diagnosis, and Treatment[J]. Med (Baltim).

[22] Liu Q, Wang W, Fan W (2020). Hoffa Fracture Associated with Tibial Shaft fracture and multiple ligament avulsion fractures: a Case Report[J]. Trauma Case Rep.

[23] Li Z, Chen Z, Wang X (2022). Locking plate alone or in combination with cannulated screws for Hoffa fractures: a retrospective Study[J]. Orthop Surg.

[24] Trikha V, Das S, Gaba S (2017). Analysis of functional outcome of Hoffa fractures: a retrospective review of 32 Patients[J]. J Orthop Surg (Hong Kong).

